# METTL3 Promotes Osteogenic Differentiation of Human Periodontal Ligament Stem Cells through IGF2BP1-Mediated Regulation of Runx2 Stability

**DOI:** 10.7150/ijms.90485

**Published:** 2024-02-04

**Authors:** Xuefei Sun, Xiujiao Meng, Yu Piao, Shaojie Dong, Qianqian Dong

**Affiliations:** 1 Key Laboratory of Shaanxi Province for Craniofacial Precision Medicine Research, College of Stomatology, Xi'an Jiaotong University, Xi'an, China.; 2 Clinical Research Center of Shaanxi Province for Dental and Maxillofacial Diseases, Department of Endodontics, College of Stomatology, Xi'an Jiaotong University, Xi'an, China.

**Keywords:** METTL3, hPDLSCs, Osteogenic differentiation, Runx2, m^6^A, IGF2BP1

## Abstract

N6-Methyladenosine (m^6^A) has been reported to play a dynamic role in osteoporosis and bone metabolism. However, whether m^6^A is involved in the osteogenic differentiation of human periodontal ligament stem cells (hPDLSCs) remains unclear. Here, we found that methyltransferase-like 3 (METTL3) was up-regulated synchronously with m^6^A during the osteogenic differentiation of hPDLSCs. Functionally, lentivirus-mediated knockdown of METTL3 in hPDLSCs impaired osteogenic potential. Mechanistic analysis further showed that METTL3 knockdown decreased m^6^A methylation and reduced IGF2BP1-mediated stability of runt-related transcription factor 2 (*Runx2*) mRNA, which in turn inhibited osteogenic differentiation. Therefore, METTL3-based m^6^A modification favored osteogenic differentiation of hPDLSCs through IGF2BP1-mediated *Runx2* mRNA stability. Our study shed light on the critical roles of m^6^A on regulation of osteogenic differentiation in hPDLSCs and served novel therapeutic approaches in vital periodontitis therapy.

## 1. Introduction

Periodontitis is the most prevalent oral infection, which destroys the periodontal supporting tissue and eventually leads to dentition loss^1^, ^2^. Repair and reconstruction of destructed periodontal supporting tissue rely on mechanical plaque removal and guided tissue regeneration techniques^3^. In recent years, human periodontal ligament stem cells (hPDLSCs) have brought great hope to periodontal tissue engineering due to their excellent osteogenic differentiation potential^4^, ^5^. Studies have shown that RUNX2 (runt-related transcription factor 2), as an osteoblast-specific transcription factor, plays a key role in the osteogenic differentiation of hPDLSCs^6^, ^7^. Therefore, endogenous regulation of RUNX2 is an important means to regulate the osteogenic differentiation of PDLSCs.

N6-methyladenosine is the most common modification of eukaryotic mRNA which regulates nearly every step of the RNA metabolic cycle, including splicing, export, decay, and translation^8^, ^9^. m^6^A modification is catalyzed by a methyltransferase complex including METTL3, METTL14 and their cofactors, and demethylated by ALKBH5 and FTO enzymes, forming a homeostasis^10^. Ultimately, m^6^A-binding proteins with YTH domains, including cytoplasmic proteins YTHDF1, YTHDF2, YTHDF3, IGF2BP1, and nuclear protein YTHDC1, mediate mRNA stability and translation to regulate downstream biological effects^11^, ^12^. Recently, the insulin-like growth factor-2 mRNA binding protein (IGF2BP) family has been reported as a novel m6A reading protein that directly recognizes m6A mRNA modifications, thereby recruiting cofactors including Human antigen R (HuR) and matrix protein 3 (MATR3). The IGF2BP protein family consists of three main members: IGF2BP1, IGF2BP2 and IGF2BP3. Functionally, IGF2BPs mainly recognizes and binds mRNA through KH domain, thus enhancing the stability of m6A-modified mRNA and improving the translation efficiency of mRNA, thus regulating various physiological and pathological functions. IGF2BP1 has been identified to play an important role in the regulation of mRNA targets such as CPT1A, E2F1, PEG10, c-MYC, and CCL5 in an m6A-dependent manner^13^-^16^. Zhou et al. reported that METTL3 promotes osteoblast differentiation of bone marrow mesenchymal stem cells (BMSCs) through IGF2BP1-mediated *Runx2* mRNA stabilization to regulate osteogenic responses^17^. METTL14 promotes postmenopausal osteoporosis through m^6^A-guided epigenetic inheritance by regulating* SMAD1* mRNA stability in an IGF2BP1 dependent manner^18^. Whether m^6^A methylation plays a role in regulating osteogenic differentiation of hPDLSCs and its molecular mechanism are still unclear.

In the current study, we revealed a significant increase of METTL3 and m^6^A levels in hPDLSCs undergoing osteogenic differentiation. Our results further verified that METTL3-mediated m^6^A RNA methylation induced hPDLSCs osteogenic differentiation via targeting *Runx2* mRNA stability. Taken together, our findings elucidated the regulatory role of METTL3-mediated m^6^A RNA methylation in the osteogenic differentiation of hPDLSCs and afforded a novel potential molecule target for periodontitis therapy.

## 2. Materials and methods

### 2.1. Cell culture

The hPDLSCs were isolated and cultured according to the previous study^19^, ^20^. The healthy third molars or orthodontically treated premolars were extracted after obtaining informed consent from 18-22-year-old healthy donors. After adequate cleaning of the teeth, periodontal ligament tissue was scraped from the middle third of the root surface, digested with collagenase, and cultured in complete medium containing a-MEM (Hyclone, USA) and 10% fetal bovine serum (FBS) (GIBCO, Invitrogen, USA) at 37℃ in 5% CO_2_. The culture medium was changed every 3 days, and hPDLSCs between passages 3 and 6 were used for subsequent experiments. The medium was refreshed every 3 days, and hPDLSCs between passage 3 and 6 were used in the following experiment.

### 2.2. Overexpression and knockdown of genes

The METTL3 gene was transfected into cells for overexpression and knockdown by lentivirus (Genepharma, Hangzhou, China). Overexpression and knockdown of the indicated genes were transfected into hPDLSCs using Lipofectamine 2000 (Invitrogen, Carlsbad, USA) with overexpression plasmids or siRNA, and analyzed 48-72 h later, followed by analysis 48-72 h later. The selected sequences for siRNA and shRNA knockdown as follow: sh-METTL3: 5′-GCTACCTGGACGTCAGTATCT-3′. sh-control: 5′-ACGTGACACGTTCGGAGAATT-3′. si-IGF2BP1: 5′-AUGAAACAUAACUUUCUUGUU-3′. si-control: 5′-TTCTCCGAACGTGTCACG A-3′.

### 2.3. RNA purification and quantitative real‑time PCR (qPCR)

Total RNA was isolated by using TRIzol reagent (Invitrogen, USA) from the samples according to the manufacturer's instructions. RNA was reverse transcribed into first‑strand cDNA using the PrimeScript™ RT reagent kit (Takara, Japan). Amplification was performed using 7500 Real-Time PCR System (Applied Biosystems) with SYBR Green PCR Master Mix (Takara, Japan). Amplification was performed as follows: denaturation at 95 °C for 60 s, followed by 40 cycles denaturation at 95 °C for 10 s, annealing at 60 °C for 30 s, and extension at 70 °C for 5 min. The qRT‑PCR was analyzed by using the 2^-∆∆Ct^ method and normalized to GAPDH expression. Primers for qPCR are as follows (Table 1):

### 2.4. Western blot analysis

For western blot analysis, cells were washed with ice-cold PBS and lysed in lysis buffer (Beyotime Biotechnology, Shanghai, China). Then, the lysates were added to 5× loading dye and separated by 10% SDS-PAGE, and then transferred to PVDF membranes (Millipore, Germany). The PVDF membranes were blocked in 5% fat-free dry milk for 1 h and then incubated with primary antibodies (1:1,000; Abcam, UK) against METTL3 (ab195352), RUNX2 (ab192256), OSX (ab209484), METTL14 (ab220030), WTAP (ab195380), IGF2BP1 (ab290736), Tubulin (1:1,000; ab6046), and Lamin B (1:1,000; ab133741) overnight at 4 °C. The PVDF membranes were incubated with secondary antibodies (Proteintech, USA) at RT for 1 h, and detected by odyssey chemiluminescence, and quantified by using the Image Studio software (LI-COR Biosciences, USA).

### 2.5. Alizarin red staining

The osteogenic differentiation of hPDLSCs was induced in the osteogenic differentiation medium for 3 weeks. The medium of osteogenic differentiation was composed of α-MEM (Hyclone, USA), 10 mM β-glycerophosphate (Sigma, USA), 10% FBS (GIBCO, USA), 0.1 μM dexamethasone (Sigma, USA), and 50 μg/mL of ascorbic acid (Sigma, USA). The cells after osteogenic differentiation were fixed with 4% paraformaldehyde for 15 min and rinsed with ddH_2_O. Then the cells were stained with 0.1% alizarin red staining (ARS; pH 4.2) at RT for 10 min. Subsequently, cells were washed with deionized water to wash away unbound Alizarin Red. To quantify ARS, 10% cetylpyridinium chloride was added to the well plate to fully dissolve and quantitatively analyzed under a spectrophotometer at 562 nm.

### 2.6. Oil red O staining

The adipogenic differentiation of hPDLSCs was induced in the adipogenic differentiation medium for 4 weeks. The medium of adipogenic differentiation was composed of α-MEM (Hyclone, USA), 10% FBS (GIBCO, USA), 0.5 mM IBMX, 200 μM indomethacin, 1 μM dexamethasone, and 10 μg/mL of insulin (Sigma, USA). The cells after adipogenic differentiation were fixed with 4% paraformaldehyde for 15 min and rinsed with ddH_2_O. Then the cells were stained with 0.1% oil red O staining for 30 min at room temperature.

### 2.7. Flow cytometry analysis

The immunophenotype of hPDLSCs was detected by flow cytometry according to the manufacturer's instructions (BD Bioscience, USA). The following antibodies were used: MSC positive markers (CD105-PE, 146-PE, and CD29-PE), the hematopoietic marker (CD34-PE) and the leukocyte marker (CD45-PE). hPDLSCs were digested by trypsin and the cell density was adjusted to 1*10^7^ in PBS solution. Then incubate with the antibodies for 30 min at 37°C in the dark. Unbound antibodies were washed with PBS and detected by flow cytometry.

### 2.8. Cell Counting Kit-8 (CCK-8) and Clonal Formation Unit

Cell proliferation was assessed by CCK-8 following the manufacturer's instructions. Briefly, we adjusted the cell density to 3000 cells/well seeded in 96-well dishes. The 96-well dishes were then incubated in a 37°C, 5% CO_2_ incubator. Cell samples were collected every day, and then 100 µl of CCK-8 solution was added to each well, and the absorbance at 450 nm was measured with a microplate reader.

HPDLSCs were limitedly diluted and seeded into 10 cm dishes at 200 cells/well. The cell culture medium was refreshed every 3 days. After 14 days, the stem cells were washed with PBS, fixed with 4% paraformaldehyde for 15 minutes, and then stained with 0.1% crystal violet to calculate the formation of cell clone-forming units.

### 2.9. m^6^A Dot Blot

Total RNAs were extracted by using TRIzol reagent (Invitrogen, USA) and then extracted and purified by an mRNA purification kit (TOYOBO, Japan) according to the manufacturer's protocol. The extracted mRNAs were directly spotted onto a Hybond-N^+^ membrane (Beyotime, Shanghai, China) and then UV-crosslinked to the Hybond-N^+^ membrane by a Stratalinker 2400 UV crosslinker (UVP, CA, USA). The membrane was washed with Tris-buffered saline with Tween 20 (TBST) for three time to remove unbound mRNAs and then blocked with 5% fat-free dry milk for 1 h at room temperature. Next, the membrane was incubated with N6-mA antibody (1:500, Synaptic Systems, Germany) at 4℃ overnight followed by HRP-conjugated anti-rabbit IgG (1:500, Synaptic Systems, Germany) for 1 h at room temperature. Finally, the membrane was mixed with Thermo ECL Signal Western Blotting Detection Reagent (Thermo Fisher Scientific, Waltham, USA) and analyzed with Image Studio software (LI-COR Biosciences, USA).

### 2.10. m^6^A Quantification

Quantification of m^6^A was measured by an m^6^A RNA methylation assay kit (Abcam, Cambridge, UK) according to the manufacturer's instructions. Briefly, positive control (PC), negative control (NC), and 200 ng isolated mRNA were added to each well with the capture antibody and then detected the percentage of m^6^A in the sample. After the RNA reacted with the substrate, the absorbance was determined with an enzyme-linked instrument at 450 nm. The formula for calculating the percentage of m^6^A in RNA is as follows: (S=200ng, P=1ng)



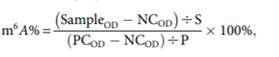



S: amount of input of RNA sample (200ng), P: amount of input of positive control (1ng), PC: positive control.

### 2.11. RNA stability assay

Cells were plated in 6-well plates and transfected with desired plasmid, siRNA or virus as described above. Cells were treated with 5 μg/ml actinomycin D (Sigma, USA) to inhibit RNA transcription and collected at indicated time points (0, 3, 6 h) to assess mRNA degradation. The total RNA was extracted and analyzed by qRT-PCR.

### 2.12. RNA agarose gel electrophoresis

Agarose (1g) was added to an erlenmeyer flask containing 90 mL of DEPC water, and heated it completely dissolve before adding 10 ml of 10x TAE buffer. Then pour the slightly cooled gel into the glue tank to solidify for later experiments. 4 μl of total RNA, 5 μl of 1×TAE buffer, and 1 μl of 10X Gel Red loading buffer were mixed and added to the gel well. RNA electrophoresis was performed from the negative electrode to the positive electrode at a voltage of 100V for about 30 minutes. The RNA gel was then placed on an Odyssey detector to observe the electrophoresis results.

### 2.13. Methylated RNA immunoprecipitation (MeRIP)-PCR

The Magna MeRIP Kit (Millipore, Massachusetts, USA) was used following the manufacturer's protocol to detect m^6^A modification on genes of interest. Briefly, 100 μg of total RNA was mixed with 100 μL RIP lysis buffer on ice for 5 min and then incubated with anti-m^6^A antibody (Synaptic Systems, Germany)- or IgG (CS200621, Millipore)-conjugated beads in 500 μl IP buffer supplemented with RNase inhibitors at 4 °C overnight. Methylated RNA was immunoprecipitated with beads, and purified with RNeasy kit (Qiagen, Germany). Then, RNA samples were subjected to reverse transcription and RT-PCR as previously described.

### 2.14. Luciferase Reporter Assay

Luciferase assay was performed using luciferase assay reagent reporter and lysis buffer (Promega, USA) according to the manufacturer's protocol. Briefly, the pmirGLO-YAP luciferase reporter by ligating YAP CDS to the mutiple cloning site (MCS) site. The wild-type and sh-METTL3 cells were transfected with YAP-luciferase reporter plasmid with Lipofectamine 2000 reagent (Invitrogen, USA). After transfection for 24 h, cells were analyzed with the Dual Luciferase Assay system (Promega, USA). Firefly luciferase (F-luc) activity was normalized to Renilla Luciferase (R-luc) to appraise reporter translation efficiency.

### 2.15. Protein co-immunoprecipitation (Co-IP)

Protein co-immunoprecipitation was performed using Dynabeads™ Co-Immunoprecipitation Kit (Thermo Fisher Scientific, Waltham, USA) according to the manufacturer's instructions and previous study^21^. Briefly, cells were washed twice with ice old PBS and then lysed with NP40 lysis buffer supplement with protease inhibitor on ice. After the protein was sonicated and centrifuged at 4 °C, 10% supernatant was collected as input. Remaining supernatant was incubated with indicated IP antibody or IgG-conjugated protein A/G Magnetic Beads in IP buffe supplemented at 4 °C overnight. Bound protein was immunoprecipitated with magnetic beads and treated with elution buffer. The recovered proteins were washed 3 times and analyzed by Western blotting.

### 2.16. Immunofluorescent staining

Cells were seeded on glass slides and fixed in 4% paraformaldehyde for 15 minutes, then permeabilized in 0.5% Triton X-100 for 10 minutes. The samples were then treated with 2N HCl for 15 minutes and neutralized with 0.1 M sodium borate buffer pH 8.5 for 15 minutes. Then, the cell slides were blocked in blocking solution (100 mg/ml RNaseA in PBS with 5% serum) for 2 h, and then N6-mA antibody (1:200, Synaptic Systems, Germany), RUNX2 (1:200, Abcam, ab192256), METTL3 (1:200, Abcam, ab195352) was added dropwise to the samples for incubation. Samples containing primary antibodies were incubated at 4°C overnight. The next day, samples were incubated with species appropriate secondary antibodies conjugated to Alexa Fluor 488 or Cy3 (1:200; Proteintech, USA) for 1 h in a humidified chamber at 37°C. Finally, slides were rinsed with PBS, nuclei were stained with DAPI, and image capture was observed under a fluorescence microscope (Olympus, Japan).

### 2.17. Statistical Analysis

Statistics were performed using Graphpad Prizm 8 (GraphPad Software Inc.,San Diego, CA, USA). Statistically significant differences were determined using a two-tailed Student's t-test or ANOVA. Data represented the mean ± SD of at least n=3 independent experiments. *p<0.05, ns, not significant.

## 3. Results

### 3.1. Identification of hPDLSCs

**Figure 1:**
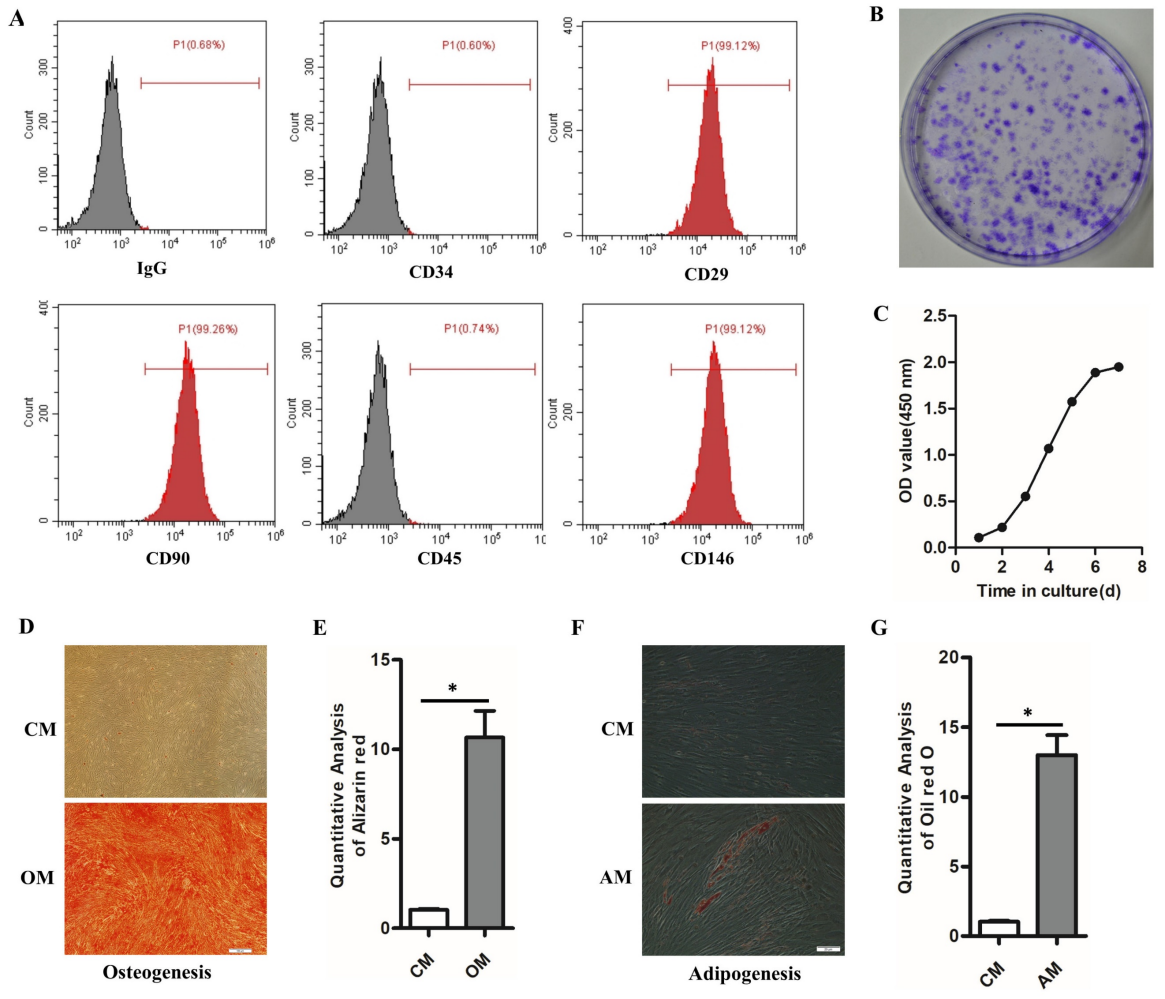
Identification of hPDLSCs (A) Cell markers CD29, CD34, CD45, CD90 and CD146 expression was measured by flow cytometry. (B) The self-renewal ability of hPDLSCs was detected by colony-forming unit staining. (C) The proliferation curve was detected by spectrophotometry. (D) The osteogenesis capabilities of hPDLSCs were detected by alizarin red. Scale bars, 100 μm. (E) The quantitative analysis of alizarin red staining. (F) The adipogenesis capabilities were detected by oil red O staining. Scale bars, 20 μm. (G) The quantitative analysis of alizarin red staining and oil red O staining. OM, osteogenic medium; CM, control medium; AM, adipogenic medium. *P< 0.05.

hPDLSCs express stem cell surface molecules, and possess high proliferation rate, self-renewal and multilineage differentiation capabilities. Flow cytometry results showed that hPDLSCs highly expressed CD29, CD90, and CD146 (mesenchymal stem cell markers) but scarcely expressed CD34 (hematopoietic marker) and CD45 (leukocyte marker) (Fig. 1A). Colony formation staining and cell growth curve verified the self-renewal ability and high proliferation ability of hPDLSCs (Figs. 1B and C). The multi-differentiation ability of hPDLSCs was verified by the Alizarin Red and Oil Red O staining. The Alizarin Red S staining indicated that mineralized calcium deposits were formed in hPDLSCs after culturing for 3 weeks in OM (Figs. 1D, E). Oil red O staining result demonstrated that hPDLSCs formed lipid droplets after 4 weeks of adipogenic induction (Figs. 1F, G). By contrast, few mineralized calcium deposits and lipid droplets were observed in the control groups (Figs. 1D-F). These data manifested that the isolated hPDLSCs hold the abilities of self-renewal and multi-differentiation.

### 3.2. The m^6^A methylase METTL3 is upregulated with the osteogenic differentiation of hPDLSCs

**Figure 2:**
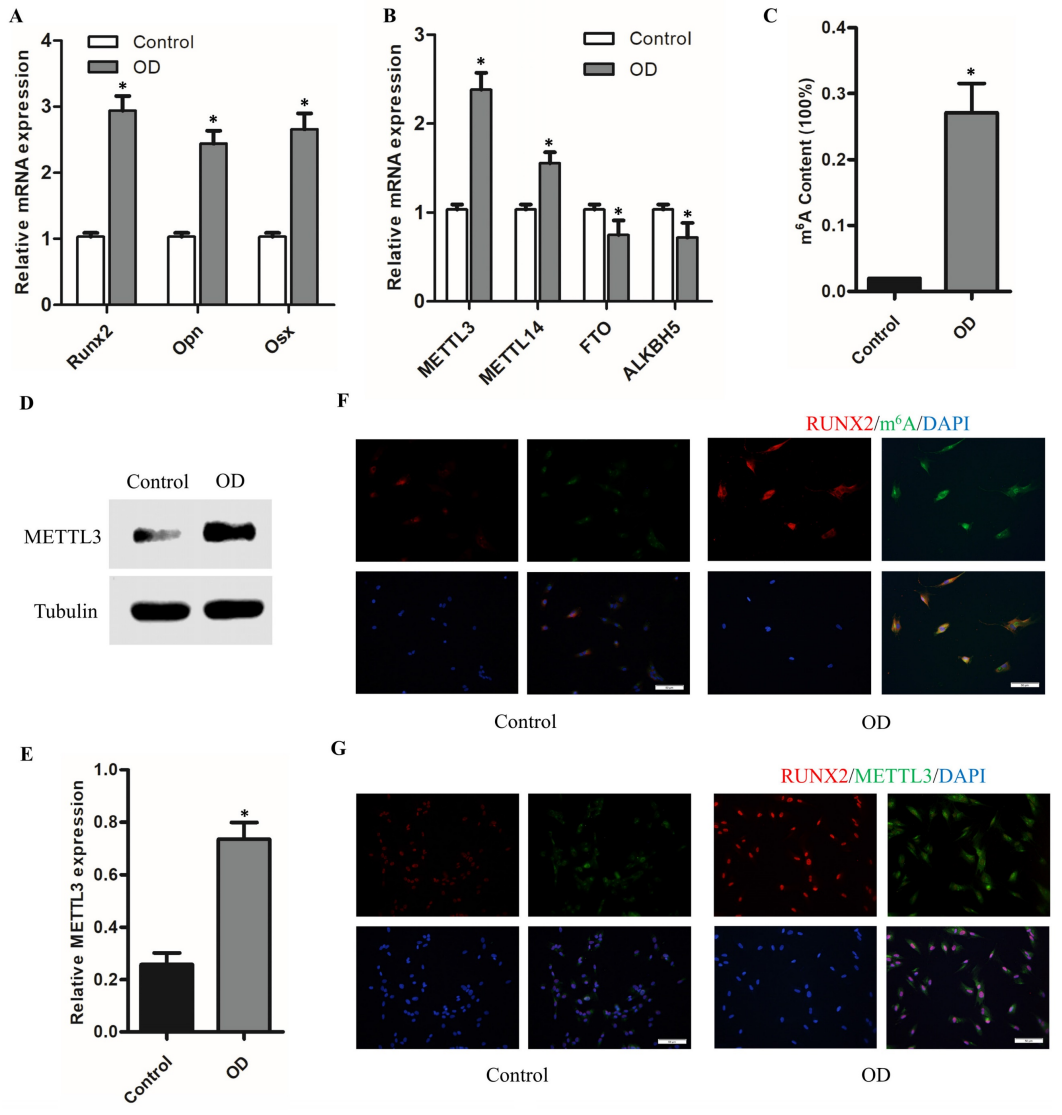
Increased m^6^A modification level together with the elevated expression of methylase METTL3 during the osteogenic differentiation of hPDLSCs. (A) The osteogenic-related genes level was detected by qPCR after osteogenic differentiation of hPDLSCs. (B) The expression of methylase (METTL3 and METTL14) and demethylase (FTO and ALKBH5) was detected by qPCR. (C) Quantification of m^6^A RNA methylation was determined by m^6^A ELISA kit. (D) The expression of methylase METTL3 was detected by Western Blotting. (E) The quantitative analysis of METTL3 expression. (F) The RUNX2 and m^6^A level was measured by immunofluorescent staining. Scale bars, 50 μm. (G) Immunofluorescent staining assay indicated that the protein levels of RUNX2 and METTL3 were elevated during the osteogenic differentiation of hPDLSCs. Scale bars, 50 μm. OD, osteogenic differentiation; *P< 0.05.

To understand the role of m^6^A in the osteogenic differentiation of hPDLSCs, we evaluated the expression of methylase (METTL3 and METTL14), demethylase (FTO and ALKBH5) and total methylated m^6^A RNA levels. As shown in the Figure 2A, the osteogenic-related mRNA level was remarkedly upregulated with the osteogenic differentiation of hPDLSCs. However, among the methyltransferase and demethylases, METTL3 level was remarkedly elevated with the osteogenic differentiation (Fig. 2B). Notably, the total m^6^A methylated RNA level was also increased with the osteogenic differentiation (Fig. 2C). The increased expression of METTL3 protein in hPDLSCs was further confirmed by western blotting (Figs. 2D, E). In accordance with the above results, the immunofluorescence (IF) assays results showed that m^6^A contents increased significantly with the up-regulation of RUNX2 in osteogenic-differentiation hPDLSCs (Fig. 2F). Moreover, there was a significant increase in methylase METTL3 correlated with m^6^A contents in OD group as compared with control group (Fig. 2G). Intriguingly, we also found that part of the METTL3 protein was transferred from the nucleus to the cytoplasm (Fig. 2G). These results evidenced that METTL3 may be involved in the osteogenic differentiation of hPDLSCs via m^6^A modification.

### 3.3 METTL3 promote osteogenic differentiation of hPDLSCs by modulating *Runx2* mRNA stability

**Figure 3:**
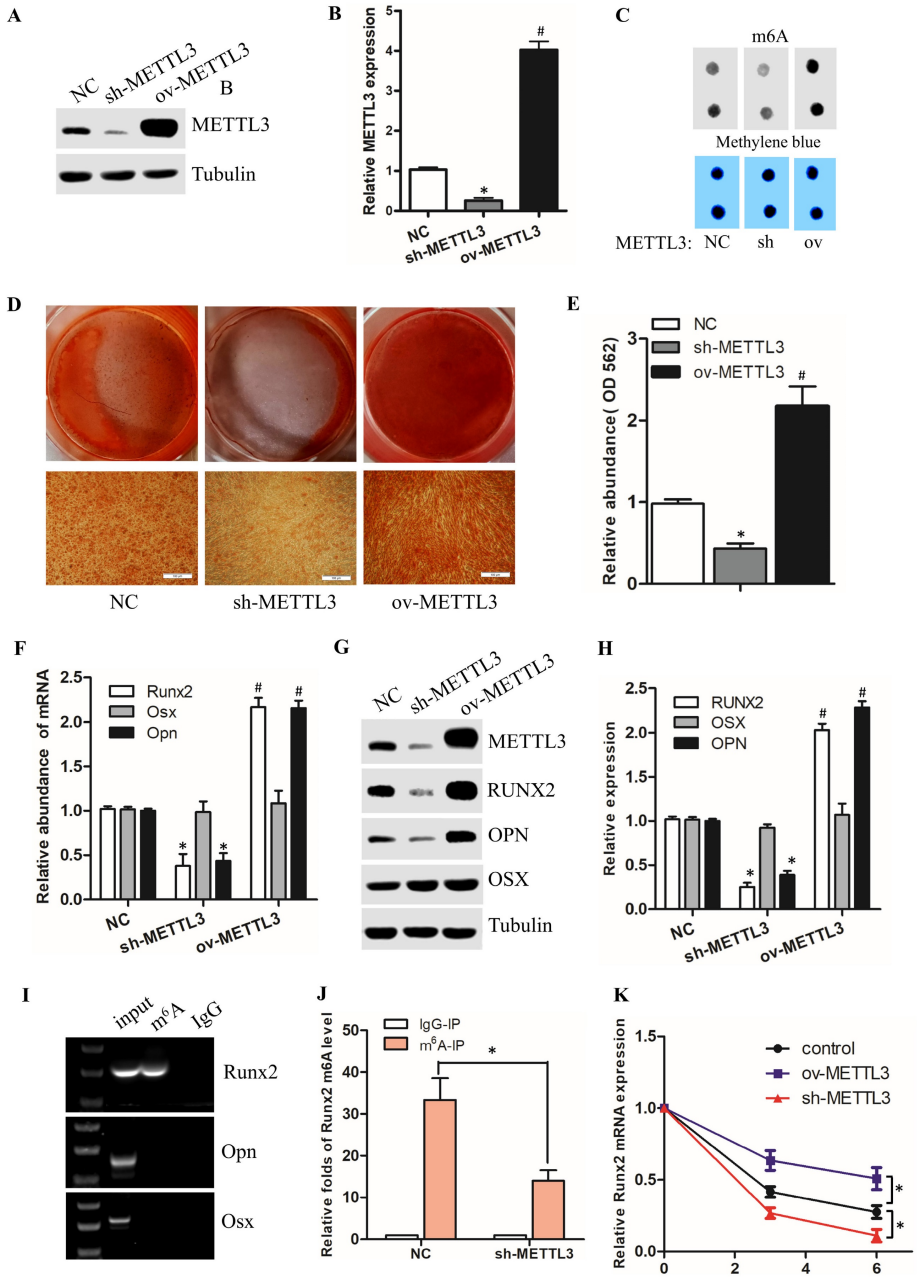
METTL3 promotes osteogenic differentiation of hPDLSCs via increasing *Runx2* RNA stability. (A) Western blot analysis of the expression of METTL3 in hPDLSCs after transfection with negative control (NC), METTL3 shRNA or METTL3. (B) Quantitative analysis of the expression of METTL3 in A. (C) The m^6^A mRNA methylation was determined by m^6^A dot blot. (D) The results of alizarin red staining in hPDLSCs that were treated as described above. (E) Quantitation of ARS absorbance measurement was performed. (F) The osteogenic biomarkers expression was detected by qPCR. (G) The expression of METTL3 and osteogenic biomarkers was detected by Western Blotting. (H) The quantitative analysis of Western Blotting in G. (I) The osteogenic biomarkers methylated m^6^A level was measured by MeRIP RT-PCR. (J) The *Runx2* methylated m^6^A level was detected by MeRIP-PCR. (K) The RNA stability assay was initiated by adding actinomycin D (an RNA polymerase II inhibitor, 5 µg/mL). The mRNA level of *Runx2* was analyzed by qPCR in Actinomycin D treated the indicated time points (0, 3, 6 h). *P< 0.05.

To verify the function of METTL3 in osteogenic differentiation of hPDLSCs, we use lentivirus to conduct gain-of-function and loss-of-function studies in hPDLSCs. As shown in Fig. 3A-B, compared to the NC group, the protein level of METTL3 was dramatically decreased in sh-METTL3 groups, and obviously increased in ov-METTL3 groups. Spontaneously, the m^6^A methylated mRNA level was repressed in sh-METTL3 groups, and elevated in ov-METTL3 groups (Fig. 3C). Moreover, alizarin red staining (ARS) was performed to investigate the osteogenic function of METTL3 in hPDLSCs. The results revealed that METTL3 knockdown markedly inhibited the osteogenic differentiation of hPDLSCs, while overexpression of METTL3 induced the opposite effects (Fig. 3D, E). In addition, the qPCR results showed that knocking down METTL3 impaired the expression of the osteogenic biomarkers (*Runx2* and *Opn*), and vice versa (Fig. 3F). The protein level of osteogenic biomarkers was detected by Western Blotting, which was consistent with the results of qPCR and Alizarin Red (Fig. 3G, H). Taken together, these findings suggest that METTL3 promotes osteogenic differentiation of hPDLSCs.

To identify the underlying mechanisms of METTL3 modulate osteogenic differentiation, we analyzed the methylated m^6^A level of osteogenic markers (*Runx2*, *Opn*, and *Osx*). The m^6^A-RIP RT-PCR revealed that among the above 3 osteogenic biomarkers, only *Runx2* had m^6^A methylation modification in hPDLSCs (Fig. 3I). Moreover, we also found that the *Runx2* methylated m^6^A level was inhibited in sh-METTL3 group compared to the control group (Fig. 3J). The above result showed that the mRNA and protein levels of *Runx2* was both dramatically up-regulated in METTL3 overexpressed hPDLSCs (Fig. 3G-H). Based on the positive correlation between *Runx2* and METTL3, we hypothesize that METTL3 promotes the osteogenic differentiation of hPDLSCs due to the increased stability of *Runx2* mRNA. To accurately evaluate the stability of *Runx2* mRNA associated with METTL3, we determined mRNA decay in actinomycin D (Act D)-treated hPDLSCs by qPCR. We observed that the *Runx2* mRNA was decreased or increased in ActD-treated hPDLSCs after knock down or overexpression of METTL3, respectively, compared with the control vectors. Taken together, the data suggested that METTL3 promotes osteogenic differentiation of hPDLSCs via increasing *Runx2* RNA stability.

### 3.4. METTL3 regulate *Runx2* mRNA stability via IGF2BP1 involvement

**Figure 4:**
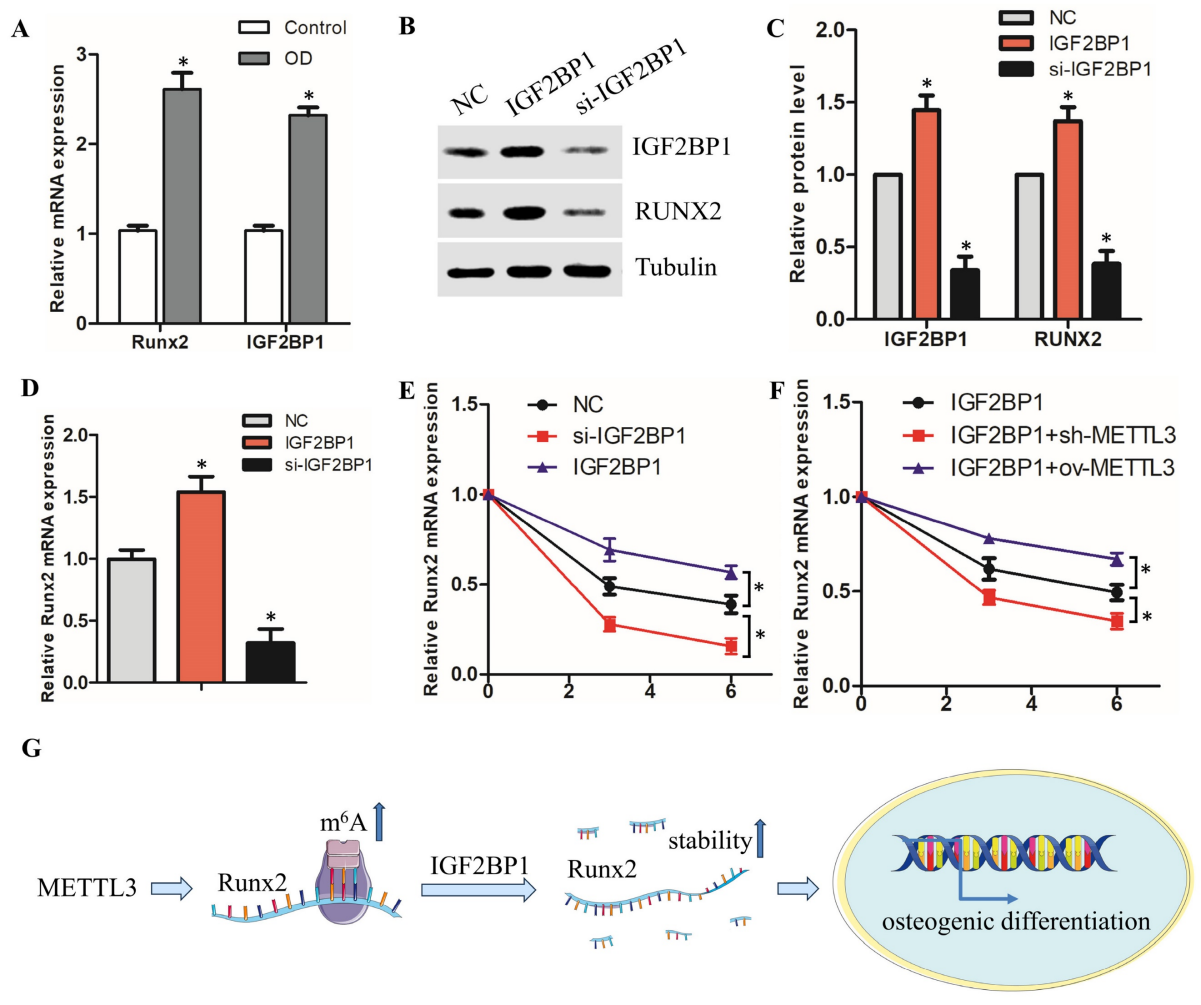
METTL3 regulate *Runx2* mRNA stability via IGF2BP1 involvement. (A) The expression of *Runx2* and IGF2BP1 was detected by qPCR after osteogenic differentiation of hPDLSCs. (B, C) Western blot analysis of the expression of IGF2BP1 and RUNX2 in hPDLSCs after transfection with negative control (NC), IGF2BP1 siRNA or IGF2BP1 plasmid. (D) The expression of *Runx2* was detected by qPCR in hPDLSCs that were treated as described above. (E) The mRNA level of *Runx2* was analyzed by qPCR in Actinomycin D (5 µg/mL) treated the indicated time points (0, 3, 6 h). (F) The RNA stability assay was performed to analyze the mRNA level of *Runx2* by qPCR in Actinomycin D (5 µg/mL) treated cells with transfected with indicated genes. (G) Schematic illustration of this study. METTL3 promotes osteogenic differentiation of human periodontal ligament stem cells through mediating *Runx2* stability via IGF2BP1-mediated m^6^A modification. OD, osteogenic differentiation; *P< 0.05.

Recent studies have shown that m^6^A mediates mRNA fates by promoting the stability of mRNA via IGF2BP1^14^-^17^. We therefore investigated whether and how the IGF2BP1 protein in hPDLSCs, which recognize the m^6^A modification to regulate *Runx2* mRNA stability. Firstly, we found that the IGF2BP1 mRNA level was remarkedly upregulated with the osteogenic differentiation of hPDLSCs (Fig. 4A). The positive correlation between *Runx2* and IGF2BP1 suggests that IGF2BP1 may mediate the decay of *Runx2* mRNA. To investigate the function of IGF2BP1 in regulating *Runx2* mRNA stability, we knocked down IGF2BP1 using si-IGF2BP1 or overexpressed the protein with a plasmid (Fig. 4B). IGF2BP1 overexpression increased, while knocking down of IGF2BP1 decreased, the mRNA and protein levels of *Runx2* in hPDLSCs, as analyzed by qPCR and Western Blotting (Fig. 4B-D). The RNA stability assay revealed that the *Runx2* RNA degradation rates after transcription inhibition was dramatically decreased or increased after overexpression or knock down of IGF2BP1, respectively (Fig. 4E). Furthermore, IGF2BP1-associated *Runx2* mRNA stability was decreased with sh-METTL3 cotransfection in hPDLSCs, but stability of *Runx2* mRNA was increased with METTL3 overexpression (Fig. 4F). These observations revealed that IGF2BP1-facilitated *Runx2* mRNA stability is regulated by METTL3-mediated m^6^A modification (Fig. 4G).

## 4. Discussion

The treatment of periodontitis aims at promoting the regeneration of alveolar bone tissue as much as possible after plaque removal^22^. Osteogenic differentiation of hPDLSCs is an important process of alveolar bone formation^23^. However, the amount of hPDLSCs in periodontal tissue is very limited. Identifying novel methods to improve the osteogenic differentiation ability of hPDLSCs is a key issue in periodontal tissue engineering^24^. In the current study, we clarified the molecular regulatory mechanism of m^6^A modification and function of *Runx2*, regulated by METTL3 and YTHDFs proteins, in the regulation of hPDLSCs osteogenic differentiation. The presented findings indicate that m^6^A modification in hPDLSCs is a novel target for a potential periodontitis therapy.

m^6^A is a conserved post-transcriptional modification with broad functional effects on homeostasis, accounts for more than 60% of all RNA modifications in eukaryotes, and its disturbance may lead to dysfunction or disease^25^, ^26^. The dynamic modification of m^6^A is regulated by methylases and demethylases. Knockout of the methylase Mettl3 has been reported to lead to increased osteoporosis in mouse^27^. However, prior to the present study, it was unclear whether the m^6^A methylase METTL3 exerted effects on the osteogenic differentiation of hPDLSCs. For the first time, we found that the methylases METTL3 and m^6^A increased synchronously with the osteogenic differentiation of hPDLSCs. Furthermore, overexpression of METTL3 upregulates *Runx2* levels by methylating the transcript through m^6^A modification and promotes osteogenic differentiation of hPDLSCs, and vice versa (Figure 3). It is worth mentioning that we also silenced FTO or ALKBH5 by siRNA, both of which could slightly promote *Runx2* expression (data not shown). Given that FTO, ALKBH5 and METTL3, METTL14 are the two ends of the m6A "seesaw", they further verified the function of METTL3 in promoting osteogenic differentiation of hPDLSCs.

The m^6^A modification requires interaction with binding proteins to exert biological functions^28^. It has been previously reported that YTHDF1 binding proteins promotes FZD5 mRNA translation in an m^6^A-dependent manner to facilitates the progression of hepatocellular carcinoma^29^. Zhang et al. revealed that silencing METTL3 inhibits osteoblast differentiation of MC3T3-E1 cells by stabilizing Smurf1 and Smad7 mRNA transcripts via YTHDF2 involvement^30^. However, it also remains elusive whether these binding proteins function in the osteogenic differentiation of hPDLSCs. As a core transcription factor of osteoblasts, RUNX2 plays an indispensable role in osteogenic differentiation^31^. In present study, we found that METTL3 promote osteogenic differentiation of hPDLSCs via increasing *Runx2* RNA stability, further observations revealed IGF2BP1-reduced *Runx2* mRNA degradation is regulated by METTL3-mediated m^6^A modification. We also found that the methylation of Opn and Osx was not detected in periodontal ligament stem cells, suggesting that the changes in Opn expression after METTL3 knockdown may be regulated by the RUNX2 transcription factor. Our observations indicate that METTL3 regulates the degradation efficiency of *Runx2* mRNA through IGF2BP1, thereby clarifying the regulatory molecular mechanism of *Runx2* m^6^A modification involved in the osteogenic differentiation of hPDLSCs from a novel perspective.

Collectively, our study clarified that METTL3 promotes the osteogenic differentiation of hPDLSCs in an m^6^A-dependent manner, acts at least partially by facilitating *Runx2* expression through mechanisms with IGF2BP1-mediated *Runx2* mRNA stability (Fig. 4G). These findings provide a novel perspective for effective therapeutic strategies for periodontitis.

## Figures and Tables

**Table 1:** Primers for qPCR.

Gene	Forward primer		Reverse primer
METTL3		5′-CAAGCTGCACTTCAGACGAA-3′	5′-GCTTGGCGTGTG GTCTTT-3′
METTL14		5′-GTCTTAGTCTTCCCAGGATTGTTT-3′	5′-AATTGATGAGATTGCAGCACC-3′
ALKBH5		5′-ACTGAGCACAGTCACGCTTCC-3′	5′-GCCGTCATCAACGACTACCAG-3′
FTO		5′-GACCTGTCCACCAGATTTTCA-3′	5′-AGCAGAGCAGCATACAACGTA-3′
Runx2		5′-GGGTAAGACTGGTCATAGGACC-3′	5′-CCCAGTATGAGAGTAGGTGTCC-3′
Osx		CCTCTGCGGGACTCAACAAC	AGCCCATTAGTGCTTGTAAAGG
Ocn		5′-GGGTAAGACTGGTCATAGGACC-3′	5′-CCCAGTATGAGAGTAGGTGTCC-3′
Alp		5′-CATGCTGAGTGACACAGACAAGAA-3′	5′-ACAGCAGACTGCGCCTGGTA-3′
GAPDH		5′-GGTCGGAGTCAACGGATTTG-3′	5′-GGAAGATGGTGATGGGATTTC-3′
			
